# Circulating Neurofilament Light Predicts Cognitive Decline in Patients With Post-stroke Subjective Cognitive Impairment

**DOI:** 10.3389/fnagi.2021.665981

**Published:** 2021-05-17

**Authors:** Jian-Hong Wang, Jie Huang, Fu-Qiang Guo, Fang Wang, Shu Yang, Neng-Wei Yu, Bo Zheng, Jian Wang

**Affiliations:** ^1^Department of Neurology, The Affiliated Hospital of University of Electronic Science and Technology, Sichuan Provincial People’s Hospital, Chengdu, China; ^2^Innovation Center of Nursing Research, West China Hospital, Nursing Key Laboratory of Sichuan Province, Sichuan University, Chengdu, China; ^3^Department of Neurology, Ya’an People’s Hospital, Ya’an, China

**Keywords:** acute ischemic stroke, neurofilament light, biomarker, prognosis, subjective cognitive impairment (SCI)

## Abstract

**Background:**

Subjective cognitive impairment (SCI) is common after acute ischemic stroke and adversely affects the quality of life. SCI is associated with an increased risk of developing mild cognitive impairment and dementia. Identifying biomarkers which could predict long-term cognitive outcomes of post-stroke SCI is of importance for early intervention. This study aims to investigate the association between circulating neurofilament light (NfL) and long-term cognitive function in patients with post-stroke SCI.

**Methods:**

This longitudinal study recruited 304 patients with post-stroke SCI, and serum NfL levels were determined at baseline. These patients were followed up for 12 months for the observation of cognitive change. Cognitive performances were assessed by a Chinese version of the Telephone Interview of Cognitive Status-40 (TICS-40) scale.

**Results:**

The patients were divided into a progression group (as determined by decreased TICS-40 scores) and a stable group (as determined by increased or unchanged TICS-40 scores). The progression group had significantly higher serum NfL levels than the stable group at baseline. Serum NfL levels were predictive for longitudinal cognitive decline during follow-up.

**Conclusion:**

These findings imply that circulating NfL could predict the long-term cognitive change of patients with post-stroke SCI.

## Introduction

Subjective cognitive impairment (SCI) is a common complaint post-acute ischemic stroke (AIS), with a prevalence estimates ranging between 30 and 90% (Van Rijsbergen et al., 2014, 2019, 2020; Burns et al., 2019). SCI has been reported to be associated with an increased risk of progressing to objective cognitive impairment (OCI), including mild cognitive impairment (MCI) and dementia (Lee et al., 2020; Liew, 2020). Once the patients advanced to OCI stage, the quality of life would be adversely affected. Therefore, identifying post-stroke SCI individuals that are at risk of developing OCI is important for early intervention for long-term cognitive consequences. However, currently no or few reliable prognostic biomarkers are available to monitor the cognitive change of post-stroke SCI patients.

Neurofilament light (NfL) is a neuron-specific structural protein (Zetterberg, 2016), and NfL concentrations in cerebrospinal fluid could reflect the severity of neuronal damage (Khalil et al., 2018). Circulating NfL is suggested to be a reliable biomarker for monitoring the clinical trajectory of many types of neurodegenerative disease such as Parkinson’s disease (Mollenhauer et al., 2020) and Alzheimer’s disease (Mattsson et al., 2017). Recent evidence also demonstrated that NfL could serve as a prognostic marker of AIS (Tiedt et al., 2018; Pedersen et al., 2019). However, the predictive effects of circulating NfL for the long-term cognitive change of post-stroke SCI patients are not clear. In this study, we investigated the association between circulating NfL and the cognitive trajectory of post-stroke SCI patients.

## Subjects and Methods

### Patients

Inpatients with AIS from the Department of Neurology, Sichuan Provincial People’s Hospital and Ya’an People’s Hospital during May 1, 2016 and Dec 31, 2019 were screened for eligibility for this study. The patients were recruited at 1 month after the onset of AIS, when the patients crossed over the acute AIS stage and the symptoms were stable. The patients were included if they fulfil the following criteria: (1) aged 60 years or older, (2) with self-reported cognitive impairment, and this cognitive complaint should be subsequent to an AIS event, (3) no objective cognitive impairment was observed as determined by the Clinical Dementia Rating (CDR) scale, and (4) willing to participate in this study. The subjects were excluded if they have one of the following conditions: (1) have cognitive impairment before AIS onset, (2) have a family history of dementia, (3) have psychiatric disorders, such as schizophrenia, bipolar disorders, depression, etc., before AIS onset, (4) cannot complete the cognitive test due to hearing, language, or communicating disabilities, (5) other severe neurological diseases that may affect circulating NfL levels, such as Parkinson’s disease, Alzheimer’s disease, traumatic brain injury, etc., and (6) refused to participate in this study. Written consent for participation was obtained from the patients or their legal relatives. This study conformed with the principles of the Declaration of Helsinki and was approved by the Investigational Review Board of the Sichuan Provincial People’s Hospital and Ya’an People’s Hospital.

### Clinical Assessment and Data Collection

At baseline (1 month after stroke onset), the demographic information [including age, sex, education level, body mass index (BMI), and smoking history], medical history (including oral anticoagulants or antiplatelet drug use), and data on comorbidities (including hypertension, diabetes mellitus, hypercholesterolemia, and atrial fibrillation) were collected from the medical records. AIS was diagnosed according to the World Health Organization Multinational Monitoring of Trends and Determinants in Cardiovascular Disease criteria and was verified by magnetic resonance imaging performed within 24 h after symptom onset. The neurological deficits of the patients were examined with the National Institutes of Health Stroke Scale (NIHSS) upon admission (Goldstein and Samsa, 1997), performed by a certified stroke neurologist. AIS subtype was determined with the TOAST criteria.

### Cognitive Assessment and Patients’ Follow-Up

Assessment of cognitive function was conducted at 1 month after stroke onset. Briefly, to exclude pre-stroke SCI or OCI, cognitive functioning prior to stroke onset was assessed with a Chinese version of the short-form Informant Questionnaire on Cognitive Decline in the Elderly (IQCODE). Patients with a previous diagnosis of cognitive impairment were included based on clinical interview and an average score >4 in IQCODE. This cutoff was previously validated to achieve a high specificity but simultaneously avoiding the exclusion of a large number of patients without prior cognitive impairment (Harrison et al., 2016). As commonly used cognitive testing scales such as the Mini-mental State Examination (MMSE) and Montreal cognitive assessment (MoCa) scales require contact lime functions, a Chinese version of the Telephone Interview of Cognitive Status-40 (TICS-40) was selected to examine the current cognitive status, which was previously validated by others (Fong et al., 2009). The TICS-40 scale used in this study includes 10 variables with a maximum of 40 points. TICS-40 score ≤20 was defined as MCI, and a score ≤12 was defined as dementia according to a previous study (Fong et al., 2009). Severity of cognitive impairment was examined with the CDR scale. Patients with a CDR score ≥ 0.5 were excluded as described above. Patients were followed up for cognitive and dementia severity assessment at 12 months after the first interview. Although the TICS-40 scale was designed as a telephone interview scale, the patients in this study were interviewed face to face. The patients were interviewed by five experienced neurologists who were expert in cognitive examinations. As for the quality control of cognitive tests, 11 subjects with dementia, nine subjects with MCI, and six cognitively normal subjects were included in this study for the inter-rater reliability assessment, generating an interclass correlation coefficient of 0.933, reflecting a relatively high inter-rater reliability.

### NfL Concentration Determination and ApoE Genotyping

Blood was sampled at the first face-to-face interview, and serum was separated within 30 min after sampling and stored at −80°C until further analysis. Serum NfL was determined using the single-molecule (Simoa) array according to the manufacturer’s instructions (Li et al., 2019). Monoclonal antibodies and purified bovine NfL were used as calibrators.

ApoE genotypes (rs429358 and rs7412) were determined with the restriction fragment length polymorphism method. The PCR reactions were performed with 1 μl DNA sample, 2.0 mM Mg^2+^ (TaKaRa, Japan), 1 × GC-I buffer (TaKaRa, Japan), 1 U HotStarTaq polymerase (Qiagen, Germany), 0.2 mM dNTP (Generay Biotech, China), 2 μM multiple PCR primers (Sangon, China), and ddH_2_O in a total volume of 10 μl. The cycling program was the same as mentioned above. The digestion of endonuclease was performed with *Afl*III or *Hae*II (New England Biolabs, United States) for rs429358 or rs7412, respectively. Then, the products were analyzed with ABI3130XL sequencer (Applied Biosystems, United States).

### Statistical Analysis

Continuous variables were tested for normality, and if they were normally distributed, independent *t* test was used, but if they were not normally distributed, Mann–Whitney *U* test was used. For categorical data, two-sample tests of proportions were used to compare proportions. A logistic regression model and two linear regression models were utilized to assess the association between circulating NfL levels and cognitive outcomes. The first logistic regression model included disease progression (as indicated by TICS-40 scores ≤20 at the follow-up interview) as the dependent variable, serum NfL levels, age, sex, ApoE ε4 carrier status, education level, BMI, smoking history, antiplatelet drug use, family history of stroke, coexisting disorders including hypertension, diabetes mellitus, hypercholesterolemia, atrial fibrillation, DWI hyperintensity volume, delirium, hemorrhagic transformation, recurrent AIS, infarction locations, post-stroke anxiety, and post-stroke depression, and the baseline TICS-40 score as independent variables. We first fitted univariate models with a single candidate variable at one time. The potential risk factors as determined by a *p* < 0.2 were included in the final multivariate regression models. The final multivariate regression models were adjusted for age and sex, although these two variables were not significant in the univariate models. The second linear regression model included the cognitive status at endpoint as the dependent variable, and the independent variables were the same as described above. The third linear regression model included the change of TICS-40 scores during a 1-year follow-up as the dependent variable. As the variable “antithrombotic drug use” had collinearity with “atrial fibrillation”, it was not fitted into these regression models. The receiver operating characteristic (ROC) curve analysis was utilized to test the predictive effects of baseline serum NfL on cognitive outcomes at follow-up. Optimal sensitivity and specificity were determined *via* a non-parametric approach. The Youden index was calculated to determine the cutoff value that maximized the discriminating power of the test. Statistical analyses were conducted using SPSS statistical package, version 24 (IBM SPSS Statistics for Windows, Armonk, NY, United States), and a *p* < 0.05 was regarded as statistically significant.

## Results

### Demographic Data

**FIGURE 1:**
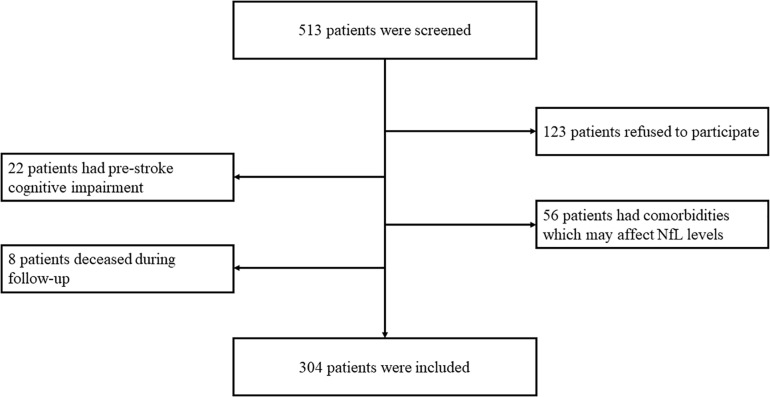
Eligibility and follow-up of patients. In total, 513 patients were screened for eligibility for participation. A total of 123 patients refused to participate, 22 patients had pre-stroke cognitive impairment, 56 patients had comorbidities which may affect serum NfL concentrations, and eight patients were deceased during follow-up. Therefore, 304 patients finally completed the follow-up.

This study screened 505 post-stroke SCI patients for eligibility for participation, among which 123 patients refused to participate, 22 patients had pre-stroke cognitive impairment, and 56 patients had co-existing disorders that may affect serum NfL concentrations; thus, 304 patients were finally recruited (Figure 1).

We divided the patients into the progression group (*n* = 49) and the stable group (*n* = 255) according to the endpoint events. Patients with decreased TICS-40 scores during follow-up were allocated into the progression group, and the others were allocated into the stable group. The stable group and the progression group had no significant differences in terms of mean age, frequencies of ApoE ε4 carriers, median education level, median BMI, frequencies of a smoking history, antiplatelet drug use, antithrombotic drug use, a family history of stroke, and frequencies of comorbidities such as hypertension, diabetes mellitus, hypercholesterolemia, and atrial fibrillation. The stable group had significantly lower frequencies of post-stroke anxiety than the progression group. However, the frequencies of post-stroke depression were comparable between the two groups. The two groups also had no significant difference in DWI hyperintensity volume and frequencies of delirium during the acute phase of AIS. No significant difference of infarction site was found between groups. Furthermore, no significant difference was observed in frequencies of hemorrhagic transformation and recurrent AIS between groups (specified in Table 1).

**TABLE 1:** Demographic data of the subjects.

Variables	Stable group (*n* = 255)	Progression group (*n* = 49)	*P* value
Age, mean (SD)	64.86 (9.37)	65.18 (8.61)	0.318^a^
Female, number (%)	107 (41.96)	23 (46.94)	0.532^b^
ApoE ε4 carriers, number (%)	34 (13.33)	5 (10.20)	0.647^b^
Education, year, median (IQR)	11 (6–15)	9 (4-14)	0.417^c^
BMI, median (IQR)	24.38 (23.07–25.61)	24.49 (23.02–25.40)	0.558^c^
Smoking history, number (%)	19 (7.45)	9 (18.37)	0.027^b^
Antiplatelet drug use, number (%)	35 (13.73)	5 (10.20)	0.647^b^
Antithrombotic drug use, number (%)	15 (5.88)	4 (8.16)	0.523^b^
Family history of stroke, number (%)	15 (5.88)	3 (6.12)	1.000^b^
**Comorbidities**			
Hypertension, number (%)	86 (33.73)	18 (36.73)	0.743^b^
Diabetes mellitus, number (%)	41 (16.08)	8 (16.33)	1.000^b^
Hypercholesteremia, number (%)	23 (9.02)	5 (10.20)	0.788^b^
Atrial fibrillation, number (%)	15 (5.88)	4 (8.16)	0.523^b^
Post-stoke anxiety, number (%)	17 (6.67)	8 (16.32)	0.031
Post-stroke depression, number (%)	14 (5.49)	5 (10.20)	0.174
DWI hyperintensity volume, ml (SD)	28.30 (9.24)	29.37 (8.02)	0.255^a^
**Infarction region^d^**			
Cerebral lobe, number (%)	44 (17.25)	8 (16.32)	0.532^b^
Cerebral white matter, number (%)	41 (16.08)	5 (10.20)	0.206^b^
Striatocapsule, number (%)	179 (70.20)	36 (73.47)	0.392^b^
Thalamus, number (%)	8 (3.14)	4 (8.16)	0.110^b^
Cerebellum, number (%)	8 (3.14)	2 (4.08)	0.499^b^
Delirium, number (%)	14 (5.49)	2 (4.08)	1.000^b^
TICS-40 at baseline, median (IQR)	26 (23–29)	29 (23–32)	0.021^c^
TICS-40 at endpoint, median (IQR)	27 (23–30)	15 (10–19)	< 0.001^c^
NIHSS at baseline, median (IQR)	12 (7–17)	12 (5–15)	0.297^c^
NIHSS at endpoint, median (IQR)	6 (2–8)	5 (1.5–7)	0.694^c^
**Stroke etiology**			
Atherothrombotic, number (%)	218 (85.49)	39 (79.59)	0.287^b^
Cardioembolic, number (%)	15 (5.88)	4 (8.16)	0.523^b^
Lacunar, number (%)	11 (4.31)	6 (12.24)	0.039^b^
Unknown, number (%)	11 (4.31)	0 (0.00)	0.222^b^
**Complication**			
Hemorrhagic transformation, number (%)	9 (0.04)	1 (0.02)	1.000^b^
Recurrent acute ischemic stroke, number (%)	3 (0.01)	2 (0.04)	0.185^b^

*IQR, inter-quartile range; BMI, body mass index; TICS, telephone interview of cognitive status 40; NIHSS, National Institutes of Health Stroke Scale.*

*^*a*^Unpaired *t* test.*

*^*b*^Pearson χ^2^ test.*

*^*c*^Mann–Whitney *U* test.*

*^*d*^It is notable that the infarctions may involve multiple brain regions.*

### Serum NfL Levels in AIS Patients With Subjective Cognitive Impairment

**FIGURE 2:**
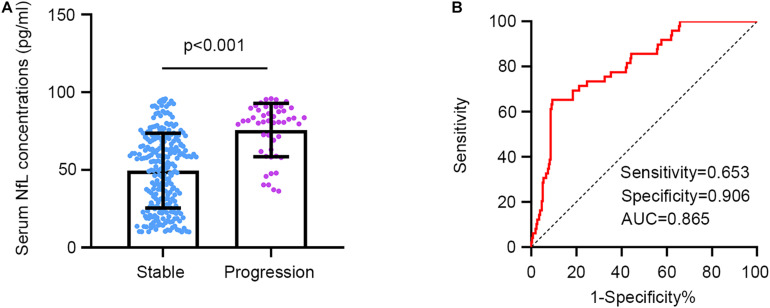
Serum NfL concentrations in patients with and without a longitudinal cognitive decline. **(A)** Serum NfL concentrations are significantly higher in the progression group in comparison with the stable group. Unpaired *t* test. **(B)** Serum NfL can differentiate the progression group from the stable group. Receiver operating characteristic analysis. Cutoff value = 79.31 pg/ml.

Serum NfL concentrations at baseline were significantly higher in the progression group (mean ± SD: 75.64 ± 17.22) in comparison with the stable group (mean ± SD: 49.47 ± 24.08, *p* < 0.001, Figure 2A). We next conducted a ROC analysis to investigate the diagnostic efficacy of serum NfL for a longitudinal cognitive decline in AIS patients with SCI. Serum NfL levels had a relatively high capacity to differentiate the progression and stable group, with a specificity of 0.906, a sensitivity of 0.653, and an area under the curve of 0.865 at a cutoff value of 79.31 pg/ml (Figure 2B).

### Association Between Serum NfL Levels and Cognitive Change During Follow-Up

We first utilized a logistic regression model to address the association between serum NfL concentrations at baseline and the progression of cognitive impairment, with the longitudinal cognitive decline (defined as a decreased TICS-40 score during follow-up) as the dependent variable and with demographic variables, medical history, and comorbidities as independent variables. In the univariate analysis, smoking history, post-stroke anxiety, and serum NfL concentrations were found to be significant predictors for a longitudinal cognitive decline. However, in the multivariate analysis, post-stroke anxiety and serum NfL remained to be significant predictors for longitudinal cognitive decline (Table 2).

**TABLE 2:** A logistic regression model to evaluate the risk factors for cognitive decline as indicated by decreased TICS-40 scores in patients with subjective cognitive impairment post-stroke.

Variables	Univariable ORs (95%CI)	*P* value	Multivariable ORs (95%CI)	*P* value
Age, year	1.004 (0.971, 1.038)	0.823	1.005 (0.967, 1.044)	0.806
Sex, male	1.224 (0.662, 2.260)	0.519	1.375 (0.679, 2.781)	0.376
ApoE ε4 carrier status	1.354 (0.502, 3.654)	0.550	0.410 (0.150, 1.121)	0.082
Education, year	0.980 (0.930, 1.033)	0.448		
BMI, kg/m^2^	0.928 (0.758, 1.137)	0.473		
Smoking history *vs*. no	0.358 (0.151, 0.846)	0.019		
Antiplatelet drug use *vs*. no	1.400 (0.519, 3.773)	0.506		
Family history of stroke *vs*. no	0.958 (0.267, 3.444)	0.948		
Hypertension *vs*. no	0.876 (0.464 to 1.656)	0.684		
Diabetes mellitus *vs*. no	0.982 (0.429 to 2.247)	0.965		
Hypercholesteremia *vs*. no	0.872 (0.315 to 2.418)	0.793		
Atrial fibrillation *vs*. no	0.703 (0.223 to 2.216)	0.548		
Anxiety *vs*. no	0.366 (0.148, 0.903)	0.029	0.183 (0.059, 0.572)	0.003
Depression *vs*. no	0.511 (0.175, 1.491)	0.219		
DWI hyperintensity volume, ml	1.013 (0.980 to 1.048)	0.449		
Cerebral lobe infarction *vs*. no	1.069 (0.469, 2.437)	0.874		
Cerebral white matter infarction *vs*. no	1.686 (0.631, 4.508)	0.298		
Striatocapsule infarction *vs*. no	0.851 (0.427, 1.693)	0.645		
Thalamus infarction *vs*. no	0.364 (0.105, 1.261)	0.111		
Cerebellum infarction *vs*. no	0.761 (0.157, 3.697)	0.735		
Delirium *vs*. no	1.365 (0.300 to 6.206)	0.687		
Hemorrhagic transformation *vs*. no	1.756 (0.217 to 14.184)	0.597		
Recurrent acute ischemic stroke *vs*. no	0.280 (0.046 to 1.720)	0.169		
TICS-40 at baseline	1.070 (0.983 to 1.165)	0.117		
Serum NfL concentrations, pg/ml	1.061 (1.040 to 1.082)	< 0.001	1.066 (1.044, 1.088)	< 0.001

*Dependent variable: cognitive decline as indicated by decreased TICS-40 scores.*

The second linear regression model included TICS-40 scores at follow-up as the dependent variable and demographic variables, medical history, and comorbidities as independent variables. TICS-40 at baseline and serum NfL were significantly associated with TICS-40 scores at endpoint (Table 3). The third linear regression model included the change of TICS-40 scores (score at follow-up – score at baseline) during follow-up as the dependent variable and demographic variables, medical history, and comorbidities as independent variables. Similarly, TICS-40 at baseline and serum NfL were significantly associated with the change of TICS-40 scores during follow-up (Table 4). This finding demonstrated that patients with lower TICS-40 and higher serum NfL concentrations at baseline were more likely to have longitudinal cognitive decline.

**TABLE 3:** A linear regression model to evaluate the risk factors for cognitive impairment as indicated by TICS-40 scores at endpoint in patients with subjective cognitive impairment post-stroke.

Variables	β unadjusted	S.E.	β adjusted	*P* value
Age, year	–0.006	0.033	–0.009	0.865
Sex, male	0.181	0.684	0.015	0.792
ApoE ε4 carrier status	0.015	0.905	0.001	0.987
Education, year	0.083	0.053	0.082	0.123
BMI, kg/m^2^	0.054	0.218	0.014	0.806
Smoking history *vs*. no	0.125	1.171	0.006	0.915
Antiplatelet drug use *vs*. no	–0.475	0.898	–0.027	0.597
Family history of stroke *vs*. no	0.187	1.421	0.008	0.895
Hypertension *vs*. no	0.075	0.653	0.006	0.908
Diabetes mellitus *vs*. no	–0.412	0.839	–0.026	0.624
Hypercholesteremia *vs*. no	–0.384	1.276	–0.019	0.764
Atrial fibrillation *vs*. no	1.109	1.318	0.046	0.401
Anxiety *vs*. no	–2.116	1.138	–0.099	0.064
Depression *vs*. no	0.090	1.318	0.004	0.945
DWI hyperintensity volume, ml	–0.080	0.051	–0.123	0.117
Cerebral lobe infarction *vs*. no	1.335	1.715	0.085	0.437
Cerebral white matter infarction *vs*. no	2.149	1.267	0.131	0.091
Striatocapsule infarction *vs*. no	1.618	1.447	0.125	0.264
Thalamus infarction *vs*. no	–1.150	1.584	–0.038	0.469
Cerebellum infarction *vs*. no	0.005	2.066	0.000	0.998
Delirium *vs*. no	1.489	1.328	0.056	0.263
Hemorrhagic transformation *vs*. no	0.937	2.037	0.028	0.646
Recurrent acute ischemic stroke *vs*. no	–3.299	2.390	–0.071	0.169
TICS-40 at baseline	0.742	0.084	0.470	< 0.001
Serum NfL concentrations, pg/ml	–0.066	0.012	–0.279	< 0.001

*Dependent variable: cognitive impairment as indicated by TICS-40 scores at endpoint.*

**TABLE 4:** A linear regression model to evaluate the risk factors for cognitive decline as indicated by a change of TICS-40 scores during follow-up in patients with subjective cognitive impairment post-stroke.

Variables	β unadjusted	S.E.	β adjusted	*P* value
Age, year	–0.006	0.033	–0.010	0.865
Sex, male	0.181	0.684	0.017	0.792
ApoE ε4 carrier status	0.015	0.905	0.001	0.987
Education, year	0.083	0.053	0.091	0.123
BMI, kg/m^2^	0.054	0.218	0.015	0.806
Smoking history *vs*. no	0.125	1.171	0.007	0.915
Antiplatelet drug use *vs*. no	–0.475	0.898	–0.030	0.597
Family history of stroke *vs*. no	0.187	1.421	0.008	0.895
Hypertension *vs*. no	0.075	0.653	0.007	0.908
Diabetes mellitus *vs*. no	–0.412	0.839	–0.029	0.624
Hypercholesteremia *vs*. no	–0.384	1.276	–0.021	0.764
Atrial fibrillation *vs*. no	1.109	1.318	0.051	0.401
Anxiety *vs*. no	–2.116	1.138	–0.110	0.064
Depression *vs*. no	0.090	1.318	0.004	0.945
DWI hyperintensity volume, ml	–0.080	0.051	–0.137	0.117
Cerebral lobe infarction *vs*. no	1.335	1.715	0.095	0.437
Cerebral white matter infarction *vs*. no	2.149	1.267	0.145	0.091
Striatocapsule infarction *vs*. no	1.618	1.447	0.139	0.264
Thalamus infarction *vs*. no	–1.150	1.584	–0.042	0.469
Cerebellum infarction *vs*. no	0.005	2.066	0.000	0.998
Delirium *vs*. no	1.489	1.328	0.063	0.263
Hemorrhagic transformation *vs*. no	0.937	2.037	0.032	0.646
Recurrent acute ischemic stroke *vs*. no	–3.299	2.390	–0.079	0.169
TICS-40 at baseline	–0.258	0.084	–0.181	0.002
Serum NfL concentrations, pg/ml	–0.066	0.012	–0.310	<0.001

*Dependent variable: cognitive decline as change of TICS-40 scores during follow-up (TICS-40 at baseline – TICS-40 at endpoint).*

## Discussion

The present study investigated the association between baseline circulating NfL levels and the longitudinal cognitive decline in a cohort of AIS patients with SCI. To our knowledge, this is the first report on the predictive effects of circulating NfL levels for the longitudinal cognitive change in AIS patients with SCI.

SCI is suggested to be accompanied by structural changes in the brain, which might be associated with an increased risk of future OCI (Hu et al., 2019). Therefore, SCI is regarded as a pre-MCI state. SCI occurs in a substantial proportion of patients with AIS, and these subjects are supposed to have increased risks of developing OCI (Sachdev et al., 2014; Van Rijsbergen et al., 2014). Furthermore, only a minor proportion of these OCI subjects belong to Alzheimer’s disease type (Sachdev et al., 2014; Van Rijsbergen et al., 2014), indicating that vascular mechanisms might also contribute to the conversion from SCI to OCI. Therefore, identifying subjects at a high risk of developing OCI is of importance for early intervention for cognitive decline. However, currently no reliable biomarker, especially blood-based biomarker, is available to determine the long-term risk of developing OCI in patients with post-stroke SCI. We found in the present study that circulating NfL (with a cutoff value of 79.31 pg/ml) can differentiate patients with a longitudinal cognitive decline from those with stable, or improved cognitive functions during follow-up.

In this study, patients with self-reported cognitive impairment post-stroke were screened, and the IQCODE scale was used to exclude pre-stroke cognitive impairment. Furthermore, patients with a CDR score more than 0 were also excluded. These will ensure that the patients were SCI rather than OCI. Patients were recruited and NfL determination was conducted at 1 month after stroke onset to exclude NfL secretion after acute neuronal damage during the acute phase of AIS, as demonstrated by previous studies. Although the patients were interviewed face to face, we used the TICS-40 which is used as a telephone interview scale, instead of MMSE and MoCa. This is because these scales require integrated motor functions, especially functions of the right upper arm. Furthermore, we only included patients aged over 60 years in order to investigate the cognitive change during the 1-year follow-up period, which is actually not long enough for assessment of cognitive change. Indeed among the 304 included subjects, 49 subjects (16.11%) developed OCI during follow-up, which is a relatively a lower prevalence in comparison with reports from previous studies (Van Rijsbergen et al., 2014).

We found in this study that circulating NfL levels were predictive for a longitudinal cognitive decline through three regression models. The first model fitted cognitive decline during follow-up as the dependent variable and found that increased serum NfL levels were associated with a higher risk of OCI. Furthermore, the linear regression models found that lower TICS-40 scores at baseline and higher serum NfL levels were associated with a worse cognitive performance at endpoint and a larger decrease of TICS-40 scores during follow-up. This study failed to figure out the association of other factors, including age and vascular risk factors such as hypertension or diabetes mellitus, with the longitudinal cognitive decline post-stroke, which is not consistent with previous studies (Jessen et al., 2020; Lee et al., 2020; Liew, 2020). This phenomenon might be attributed to the fact that the subjects in this study had relatively old age and that the follow-up period is not long enough for the observation of the contribution of these factors to cognitive decline. NfL is released from damaged neurons after acute or chronic neuronal injury conditions. It is not clear yet why circulating NfL were still in an increased level, as compared to that in healthy subjects, in AIS recovering stage, during which damaged neurons may have been wiped out. Interestingly, it is suggested that neuronal damage due to chronic inflammation may persist in a very long period after AIS onset (Martin et al., 2018). This could, to some extent, explain the increased NfL levels during the recovery stage of AIS and that increased NfL in this stage could predict long-term cognitive decline.

This study has some limitations. First, no control cohort with SCI but without AIS was included. Therefore, we could not compare the circulating NfL levels between post-stroke SCI subjects and SCI patients without AIS. Second, we could not deny that, for an observational study, the sample size is relatively small; thus, there are some inconsistencies with previous studies, as discussed above. Third, the patients were only followed up for once, thus the dynamic change of NfL during follow-up was not investigated. However, this study identified a potential prognostic biomarker for longitudinal cognitive decline in patients with post-stroke SCI. Patients with increased circulating NfL levels after stroke should be intensively monitored for delayed cognitive decline.

## Data Availability Statement

The original contributions presented in the study are included in the article/supplementary material, further inquiries can be directed to the corresponding author/s.

## Ethics Statement

The studies involving human participants were reviewed and approved by Investigational Review Board of the Sichuan Provincial People’s Hospital and Ya’an People’s Hospital. The patients/participants provided their written informed consent to participate in this study.

## Author Contributions

JW and J-HW designed the study and drafted the manuscript. F-QG and FW collected the samples and patients’ information. SY and N-WY participated in the determination of NfL. BZ conducted the statistical analysis. All authors contributed to the article and approved the submitted version.

## Conflict of Interest

The authors declare that the research was conducted in the absence of any commercial or financial relationships that could be construed as a potential conflict of interest.
